# Assessing Physical Performance in Centenarians: Norms and an Extended Scale from the Georgia Centenarian Study

**DOI:** 10.1155/2010/310610

**Published:** 2010-09-14

**Authors:** M. Elaine Cress, Yasuyuki Gondo, Adam Davey, Shayne Anderson, Seock-Ho Kim, Leonard W. Poon

**Affiliations:** ^1^Department of Kinesiology, Department of Health Promotion & Behavior, Institute of Gerontology and Ramsey Center, University of Georgia, Athens, GA 30602-6554, USA; ^2^Osaka University, Osaka 565-0871, Japan; ^3^Temple University, Philadelphia, PA 19122, USA; ^4^University of Connecticut, Storrs, CT 06269, USA

## Abstract

Centenarians display a broad variation in physical abilities, from independence to bed-bound immobility. This range of abilities makes it difficult to evaluate functioning using a single instrument. Using data from a population-based sample of 244 centenarians (*M*_Age_ = 100.57 years, 84.8% women, 62.7% institutionalized, and 21.3% African American) and 80 octogenarians (*M*_Age_ = 84.32 years, 66.3% women, 16.3% institutionalized, and 17.5% African American) we (1) provide norms on the Short Physical Performance Battery and (2) extend the range of this scale using performance on additional tasks and item response theory (IRT) models, reporting information on concurrent and predictive validity of this approach. Using the original SPPB scoring criteria, 73.0% of centenarian men and 86.0% of centenarian women are identified as severely impaired by the scale's original classification scheme. Results suggest that conventional norms for older adults need substantial revision for centenarian populations and that item response theory methods can be helpful to address floor and ceiling effects found with any single measure.

## 1. Introduction

The oldest old display a broad range and variability of physical and cognitive abilities [1–6]. The large range of performance presents a significant measurement problem to researchers. For example, about one-third of centenarians perform well cognitively, at the range of those who are in their 60s and 80s; on the other hand, about 50% of centenarians have some form of dementia, and about one-third have moderate to severe dementia [7]. Similar measurement issues are present for physical functions. Handgrip strength in the oldest varies from <5 kg to >30 kg [5, 8]. Some centenarians live independently and perform all physical and instrumental activities of daily living while others are immobile and bed bound [5, 8]. Few norms exist for physical performance among centenarians and a central problem is that current functional performance batteries display both floor and ceiling effects. 

The purpose of this paper is to (1) present normative data for centenarians on the Short Physical Performance Battery and (2) provide evidence for the validity of an extended SPPB scaling that addresses issues of floor and ceiling effects by combining data from instruments with different levels of scaling into one continuous scale developed using item response theory (IRT). For those expected to perform well, we chose to use the Short Physical Performance Battery (SPPB) [9, 10]. It has been used in several large epidemiological studies and has been shown to have predictive validity for those with moderate to high levels of mobility disability and morbidity. For those physically weak and nonambulatory participants, we chose to use items on the Physical Performance Mobility Exam (PPME) not included on the SPPB [11].

## 2. Methods

### 2.1. Participants

Participants were 244 centenarians and near centenarians (aged 98 and older) and 80 octogenarians recruited from 44 counties in northeast Georgia, with full details described elsewhere [2]. Because the study was population based, there were no exclusions although, to be included, all centenarians were required to provide blood samples. Overall, the recruitment rate (of those contacted participating) was 67.2% for centenarians and 46.0% for octogenarians. Further, our sample represents an estimated 19.6% of the entire population of centenarians in this geographic area. The GCS employed internationally established criteria in age verification [12] using convergent multiple and creditable sources and public records, such as birth and marriage certificates of the individuals as well as their offspring and relatives to create a consistent chronology. Driver's licenses, Social Security documents, census records, as well as death records of offspring are used.

### 2.2. Materials and Procedure

A complete list of measures included in the GCS appears elsewhere [13].

#### 2.2.1. Short Physical Performance Battery (SPPB)

Is a valid measure of lower extremity mobility, predictive of mortality and institutionalization in community-dwelling older adults with a broad range of abilities [9]. The SPPB consists of (1) three standing balance measures (tandem, semi-tandem, and side-by-side stands), (2) five continuous chair stands, and (3) a 2.44-meter walk. The scaling was developed by dividing the performance times on the original population Established Populations Epidemiological Studies in the Elderly (EPESE) into quartiles from 1 (the lowest) through 4 (the highest, with 0 assigned to nonperformers. The three balance tests are considered a hierarchy of difficulty when assigning a single score of zero to four for standing balance. Individuals unable to complete tasks are given the score of zero on that task. Completed tasks were assigned scores from one to four based on time, where the shortest time received the score of four. The scores were summed to get a total score ranging from zero to 12. Poor performance is a risk factor for mortality in data gathered from epidemiological studies on community-dwelling populations in their eighth and ninth decade [10].

#### 2.2.2. Physical Performance Mobility Exam (PPME)

It was developed and validated on hospitalized patients and includes lower functioning tasks in addition to those on the SPPB described above [11]. The additional tasks include (1) bed mobility to assess the ability to move from lying to sitting positions, (2) transferring from sitting on the edge of a bed to sitting in a chair, and (3) stepping up one step with or without the use of a bed handrail. This measure used a 3-level scoring system where 0 was assigned to nonperformers, and 1 was assigned to those completing without assistance in ≥10 seconds (bed mobility), with assistance (transfer), with use of handrail (step-up). 2 was assigned to those completing in <10 sec (bed mobility), without assistance (transfer), or without use of handrail (step-up).

#### 2.2.3. GCS Composite Scale (GCS)

It was developed using item response theory (IRT) methodology based on scores on the SPPB scores (using GCS cut-off values for timed tasks) along with PPME and grip strength. Participants' latent ability was estimated as a *z*-score from the difficulty of each test item and participants' responses to them. These scores were then rescaled in 11 even division points (2–12), with 1 assigned to nonperformers. (Figure S1 shows the information provided by each task as a function of latent ability. Table S1 shows time cut-offs to provide quartiles in the EPESE and the GCS data sets).

#### 2.2.4. Direct Assessment of Functional Status (DAFS)

It is a clinician-rated scale based on performance on time orientation, communication, transportation, preparing for grocery shopping, financial skills, grocery shopping, dressing and grooming, and eating [14]. Transportation, preparing for grocery shopping, and grocery shopping tasks of the DAFS were omitted due to increased physical demands and low likelihood that centenarians were currently engaged in these activities. Each activity of daily living (ADL) tasks on the DAFS was scored on a dichotomous scale based on the participant's successful completion of the functional task. The BADL score was calculated by summing the grooming, dressing, and eating scales (possible range = 0–23 points and higher scores represent higher functional status); the IADL score was calculated by summing the time orientation, communication, and financial skills scales (possible range = 0–58 points and higher scores represent higher functional status). The DAFS has been validated with community-dwelling samples [15] and older adults with dementia [14].

#### 2.2.5. Grip Strength

It was assessed using the Jamar (Detecto, Jackson, MI) hand grip dynamometer. After adjusting the handle to the second metatarsal, while sitting in a chair with the arm allowed to hang down at the side, maximal grip strength was tested three consecutive times on both the right and left hands. Peak force to the nearest tenth kilogram (0.1 kg) was calculated for each hand. Analyses use the average peak value across both hands (average values correlated *r* > .97 with values obtained from each hand.)

#### 2.2.6. Knee Extensor Strength

It was tested using a manual muscle manometer (Nichols, LaFayette IN). Positioned in a straight backed chair with the lower leg hanging freely where the foot did not touch the floor and arms were folded across the chest to avoid use of the upper body, the participant was asked to straighten the leg as forcefully as possible while administrator maintained stability. Peak force to the nearest tenth kilogram (0.1 kg) was calculated for each leg. Analyses use the average peak value across both legs (average values correlated *r* > .98 with values obtained from each leg).

### 2.3. Test Administration

Based on results from pilot testing with 10 centenarians (not included in this sample), administration of SPPB and PPME was originally tailored to reduce participant burden using a decision rule based on participant ambulatory ability. If participants could stand, only items of the SPPB and the step-up of the PPME were administered. Otherwise, if they are unable to stand, only the bed mobility and transfer tasks were administered. During testing of the current sample, it was determined that these tasks were not strictly hierarchical for this population. As a result, the protocol was changed so that all tasks were offered to all participants. In most cases for participants who were administered only one scale or the other, it was possible to recreate ability on the nonadministered test by working with data from participants administered both scales as well as detailed administration notes provided by interviewers. (Procedures for completing these partial datasets is described fully under “Missing Values” in the Supplementary Material of this paper; see Supplementary Material available online at doi: 10.1155/2010/310610.) Conclusions were not altered by whether partial cases were included or excluded.

### 2.4. Statistical Analysis

SPSS (Version 17.0, Chicago, IL), Stata 11.1 (StataCorp, College Station, TX), and MULTILOG (Scientific Software International, Lincolnwood, IL) were used for all analyses. Descriptive statistics were used to determine means and standard deviations. *T*-tests were used to compare mean differences between age groups. Pearson's *r* was used for zero-order correlations, followed by comparisons of Fisher's z-transformed values across age groups [14] and for dependent correlation coefficients [14, 16]. Item response theory was used to develop the GCS Composite Score. Significance level was set at *P* < .05.

## 3. Results

### 3.1. Comparison of Physical Performance Data across Age Groups

Table 1 presents descriptive statistics for octogenarians and centenarians. As can be seen, octogenarians have significantly higher (*P* < .001) physical performance than centenarians on leg strength, grip strength, the SPPB, the PPME, and the IRT-derived physical performance measure. Consistent with the population-based nature of this study, a higher proportion of the centenarian sample was female and institutionalized compared with the octogenarian sample, but there were no differences in race.

### 3.2. Norms from the Georgia Centenarian Study

**Figure 1:**
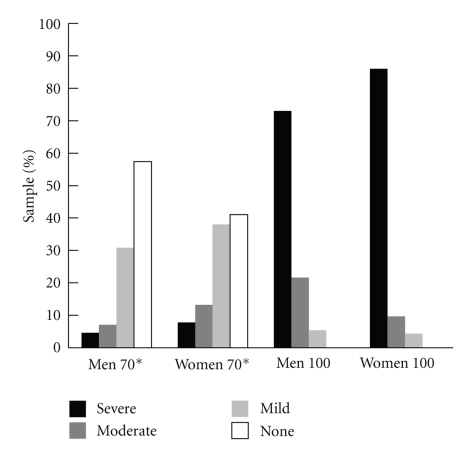
Comparison of SPPB performance categories between GCS Centenarians and EPESE 70–79 Cohort.

Figure 1 compares the proportion of the Georgia Centenarian Study sample in each of the four scoring categories reported in [5, 9]. For comparative purposes, we present our results alongside those derived from the EPESE sample for men and women aged 70 to 79 [9]. As can be seen, a large proportion of centenarians (73.0% and 86.0% of men and women, resp.) fall into the severely disabled categories whereas none could be classified as having no disability (0% for both men and women). Comparable values for octogenarians indicated that 22.2% and 30.2% of men and women, respectively, were in the most disabled category whereas 14.8% and 9.4% of men and women, respectively, were classified as having no disability. (Supplemental Table S2 provides norms by gender and age group on each performance scale. Table S3 presents the age group proportions of the sample performing at floor and ceiling for the three scales. Table S4 describes characteristics of the sample performing at the floor on each scale.)

### 3.3. Evidence for Concurrent Validity of the GCS Scale

Table 2 presents zero-order correlations among physical performance measures for octogenarians (above diagonal) and centenarians (below diagonal). For octogenarians, the GCS scale generally shows similar magnitude correlations with each of the other measures. GCS Composite scores correlate more highly with DAFS BADL scores than do SPPB scores but there are no other differences. In contrast with centenarians, GCS Composite scores correlate more highly than SPPB and PPME with DAFS BADL and IADL scores, leg extensor strength, and grip strength. The PPME is more highly correlated with DAFS BADL scores than the SPPB, but there are no other differences between the SPPB and PPME for this age group. (Figure S2 shows a scatterplot of GCS Composite scores against SPPB and PPME scores.)

### 3.4. Evidence for Predictive Validity of the GCS Scale

Predictive validity is a very important criterion for any measure of physical performance in centenarians. The distribution of time to mortality by SPPB, PPME, and GCS Composite scores are shown in Table 3 with mortality within 0–6, 7–12, 13–24, or 25+ months from interview. Both the SPPB and PPME show some irregularity in proportionality of higher performers dying earlier and low performers still alive. In sharp contrast, the GCS Composite scale shows a regular progression of mortality where no high performers died within 6 months and a more systematic stepwise proportionality of those who died at successively longer times following assessment.

## 4. Discussion

Because of the vast range of functioning observed, centenarians present unique challenges to evaluation and assessment, particularly in the context of a population-based research. We set out to provide norms for physical performance in centenarians using established scales and to demonstrate the concurrent and predictive validity of an extended scale developed through IRT using the SPPB, PPME, and grip strength.

With regard to normative functioning, severe impairment is the modal category when the SPPB instrument was used as the criterion, and no centenarians performed at the highest levels on that scale. Centenarians score significantly lower on every indicator of physical performance than octogenarians. At the same time, however, use of a measure intended for more severely impaired populations did not solve the problem. Rather, many centenarians performed at the ceiling on the PPME. Thus, of necessity, a scale that combines the information provided at each end of the continuum is essential. By combining the tasks from two psychometrically sound instruments (SPPB and PPME) and adding a measure of grip strength in order to provide information about those with the very lowest physical performance, we were able to capture a larger range of abilities, particularly among those in the lowest functioning range. Although many approaches to scaling could have been used, we adopted IRT methodology because its origins in scaling measures across disparate ability levels when underlying true values are unknown.

In terms of concurrent validity, our GCS Composite scale performed favorably compared with either the SPPB or PPME measures, correlating more highly with observed performance on BADLs and IADLs among centenarians, as well as grip strength and leg extensor strength. Equally importantly, it performed as well as these scales among octogenarians, suggesting that our methodology was sufficient to capture the wide differences in physical performance between these age groups.

Finally, the GCS Composite scale also had favorable properties in terms of predictive validity, with higher scores associated with progressively longer time to mortality. The patterning in the other scaling methods lacks the systematic pattern of longevity.

A primary limitation of this study was the missing data which resulted from the initial attempts to limit participant burden. This was addressed through statistical and field note procedures to recover a full complement of data. Likewise, it would have been desirable to have test-retest data on our instrument, but this was not generally possible due to the taxing nature of providing physical population in a study which was already divided into 5 2-hour sessions. The strengths of this study are that data from this population sampling of the oldest provides information on the order and patterning of the most commonly measured tasks. It also provides a single performance scale with negligible floor or ceiling effects. Given the incredibly rapid growth among the centenarian population, having high quality normative data available to researchers and clinicians is of the utmost importance.

## Supplementary Material

Supplementary material for this article describes analyses and procedures pertaining to: 1) handling of missing values regarding physical performance, 2) comparison of scoring cut-offs between EPESE and GCS samples, 3) physical performance scores by gender and age group, 4) distribution of extreme scores by physical performance measure, 5) characteristics of participants scoring at floor on each physical performance measure, 6) information by GCS composite score across physical performance tasks, and 7) a scatterplot of GCS composite scores with other physical performance measures.

## Figures and Tables

**Table 1:** Sample characteristics by age group.

	Octogenarians					Centenarians					
Characteristic	*N*	*M*/%	SD	Min	Max	*N*	*M*/%	SD	Min	Max	*P*-value
Age (years)^a^	80	84.32	2.78	80.53	90.06	244	100.58	2.04	98.10	108.55	.001
Female^b^	80	66.3				244	84.8				.001
Black^b^	244	17.5				80	21.3				.525
Institutionalized^b^	244	16.3				80	62.7				.001
SPPB^c^	80	5.63	3.22	0	12	244	1.46	2.19	0	9	.001
PPME^c^	80	4.48	2.01	0	6	244	2.31	2.11	0	6	.001
GCS^c^	80	9.08	3.15	2	12	244	5.18	3.08	1	12	.001
DAFS BADL^a^	77	21.23	5.25	0	23	231	16.29	8.26	0	23	.001
DAFS IADL^c^	78	47.67	17.12	0	58	235	25.74	18.28	0	58	.001
Leg strength (kg)^a^	80	11.06	7.87	0	40.05	241	5.05	5.83	0	35	.001
Grip strength (kg)^a^	80	21.49	12.22	0	63.50	243	10.32	10.54	0	60	.001

^a^ *t*-test with unequal variances.

^b^Fisher's exact test.

^c^ *t*-test with equal variances.

**Table 2:** Intercorrelations among performance measures and criterion variables for Centenarians (below diagonal) and Octogenarians (above diagonal).

	SPPB	PPME	GCS	DAFS BADL	DAFS IADL	Leg strength	Grip strength
SPPB	1.000	0.807	0.862	**0.503**	0.582	0.467	0.410
PPME	0.724	1.000	0.952	0.583	0.633	0.445	0.469
GCS	0.783	0.891	1.000	**0.630**	0.641	0.485	0.448
DAFS BADL	***0.406***	*0.487*	**0.585**	1.000	0.839	0.432	0.496
DAFS IADL	**0.533**	0.531	**0.610**	0.747	1.000	0.508	0.532
Leg strength	**0.510**	0.518	**0.581**	0.449	0.501	1.000	0.181
Grip strength	**0.461**	0.508	**0.613**	0.431	0.475	0.406	1.000

Note. Entries which share an attribute (**bold**, underline, *italics*) are significantly different within age group, *P* < .05.

**Table 3:** Distance from mortality (months) by physical performance categories.

	% of column total			
Category	0–6	7–12	13–24	25+
	SPPB			
0–3	100.0	87.5	87.0	75.9
4–6	0.0	7.5	10.9	17.2
7–9	0.0	5.0	2.2	6.9
10–12 (not observed)	0.0	0.0	0.0	0.0

	PPME			
0	57.1	27.5	45.7	28.4
1-2	16.7	32.5	21.7	12.9
3-4	16.7	20.0	15.2	27.6
5-6	9.5	20.0	17.4	31.0

	GCS			
1–4	57.1	50.0	58.7	36.2
5-6	26.2	25.0	13.0	12.1
7–9	16.7	25.0	19.6	31.9
10–12	0.0	0.0	8.7	19.8
