# Supplementary material for: Assessing Physical Performance in Centenarians: Norms and an Extended Scale from the Georgia Centenarian Study
Source: Curr Gerontol Geriatr Res. 2010 Sep 14;2010:310610. doi: 10.1155/2010/310610 (PMC2946569; doi:10.1155/2010/310610)
Supplement: Supplementary file 1 — Supplementary material for this article describes analyses and procedures pertaining to: 1) handling of missing values regarding physical performance, 2) comparison of scoring cut-offs between EPESE and GCS samples, 3) physical performance scores by gender and age group, 4) distribution of extreme scores by physical performance measure, 5) characteristics of participants scoring at floor on each physical performance measure, 6) information by GCS composite score across physical performance tasks, and 7) a scatterplot of GCS composite scores with other physical performance measures. [file 310610.f1.pdf]

Cress et al. Supplemental Information

Supplemental Statistical Analysis

Missing values

All six tasks, (3 from the SPPB and 3 from the PPME) were performed by 162 participants. Transfer from a bed to a chair and sitting up in bed (bed mobility) were assessed on 209 individuals. Two methods were used to determine probable values when data were missing. First, tester's field comments were used to verify very low functional status. When participant's inability to perform the task was confirmed a score of zero was given. For example, the field comments for two participants in the study read: "Need two persons help to walk" or "use of bed lift". In each of these cases, relevant items with missing data were recoded to a score of zero. When the field comments did not clearly validate functional status, (e.g. "Unable to do multiple sections due to frailty" or "very uncomfortable free standing"), the value was left as missing.

In order to determine the probable score for those individuals who were not administered the transfer and bed mobility tasks, scores on these items were cross-tabulated with those of all other items for participants with complete data. Two rules from this analysis emerged. Participants who could walk faster than 4.1 seconds and could step up without gripping hand rails were always able to perform the bed mobility task without assistance in < 10 seconds (score of 2) and transfer from seated position without assistance (score of 2). Missing data for bed mobility and transfer tasks was recoded to a two for participants who met these rules (n= 23). When available, videotape from interviews with the participant used to validate the imputed values.

Table S1:

Performance scoring cut-offs for two populations: Established Populations of Epidemiological Studies of the Elderly (EPESE) and the Georgia Centenarian Study.

8-foot walk	Score	EPESE	GCS
Cut-off for slowest <25%	1	≥5.7	≥9.8
26-50%	2	5.6-4.1	9.7-5.8
51-75%	3	4.0-3.2	5.7-3.8
76-100% Cut-off for fastest	4	≤3.1	≤3.8
Chair stand			
Cut-off for slowest <25%	1	≥16.7	≥23.1
26-50%	2	13.7-16.6	23.0-17.6
51-75%	3	11.2-16.5	17.6-14.5
76-100% Cut-off for fastest	4	≤11.1	≤14.4

Table S2

Raw Scores on Physical Performance Scales by Gender and Age Group

	Octogenarians		Centenarians	
Score	Men	Women	Male	Female
		SPPB		
0	7.4	11.3	46.0	53.1
1	3.7	1.9	10.8	17.9
2	3.7	11.3	8.1	9.7
3	7.4	5.7	8.1	5.3
4	11.1	5.7	5.4	3.4
5	3.7	13.2	8.1	3.4
6	22.2	9.4	8.1	2.9
7	3.7	7.6	0.0	1.5
8	14.8	13.2	2.7	1.9
9	7.4	11.3	2.7	1.0
10	7.4	7.6	0.0	0.0
11	3.7	1.9	0.0	0.0
12	3.7	0.0	0.0	0.0
		PPME		
0	7.4	7.6	27.0	38.2
1	0.0	3.8	2.7	5.8
2	11.1	15.1	18.9	12.1
3	3.7	5.7	5.4	8.7
4	11.1	0.0	10.8	14.5
5	7.4	22.6	21.6	17.9
6	59.3	45.3	13.5	2.9
		GCS Composite		
1	0.0	0.0	5.4	18.4
2	7.4	5.7	2.7	4.4
3	0.0	0.0	21.6	19.3
4	0.0	7.6	5.4	6.3
5	11.1	7.6	8.1	7.3
6	3.7	5.7	8.1	9.7
7	3.7	5.7	5.4	12.1
8	3.7	1.9	16.2	9.2
9	3.7	3.8	8.1	3.9
10	11.1	18.9	8.1	6.8
11	25.9	17.0	5.4	1.5
12	29.6	26.4	5.4	1.5

Table S3:
Distribution of sample at the extremes of instrument scales

	Zero N (%)	Floor N (%)	Ceiling N (%)	Range
PPME	95 (29.3)	15 (4.6)	51 (15.7)	0-6
SPPB	135 (41.7)	43 (13.3)	1 (0.3)	0-12
GSC Composite	40 (12.3)	15 (4.6)	27 (8.3)	1-12

PPME = Physical Performance Mobility Exam; SPPB = Short Physical Performance Battery; GCS Composite = Georgia Centenarian Study Composite scale

Table S4:
Characteristics of Participants Scoring Zero on Each Performance Measure

	SPPB	PPME	GCS Composite
N	135	95	40
Age	100.3 (4.1)	100.4 (4.2)	101.1(2.2)
MMSE	12.3 (8.5)	10.3 (8.3)	6.7(8.19
GDRS	4.7 (1.6)	7.0 (5.0)	5.4(1.6)
Grip (Kg)	6.9 (9.9)	3.6 (4.2)	0
Leg (Kg)	2.2 (3.7)	1.4 (3.5)	0.23(1.2)
BADL	12.6 (9.0)	10.6 (9.0)	5.7 (7.9)
IADL	16.7 (15.8)	13.0 (13.9)	8.3 (13.1)

SPPB = Short Physical Performance Battery; PPME = Physical Performance Mobility Exam; GCS Composite = Georgia Centenarian Study Composite scale; GDRS = Global Deterioration Rating Scale; BADL = DAFS Basic Activities of Daily Living; IADL = DAFS Instrumental Activities of Daily Living

Figure S1

Information by GCS Composite Score (z-score scaling) for Each Task

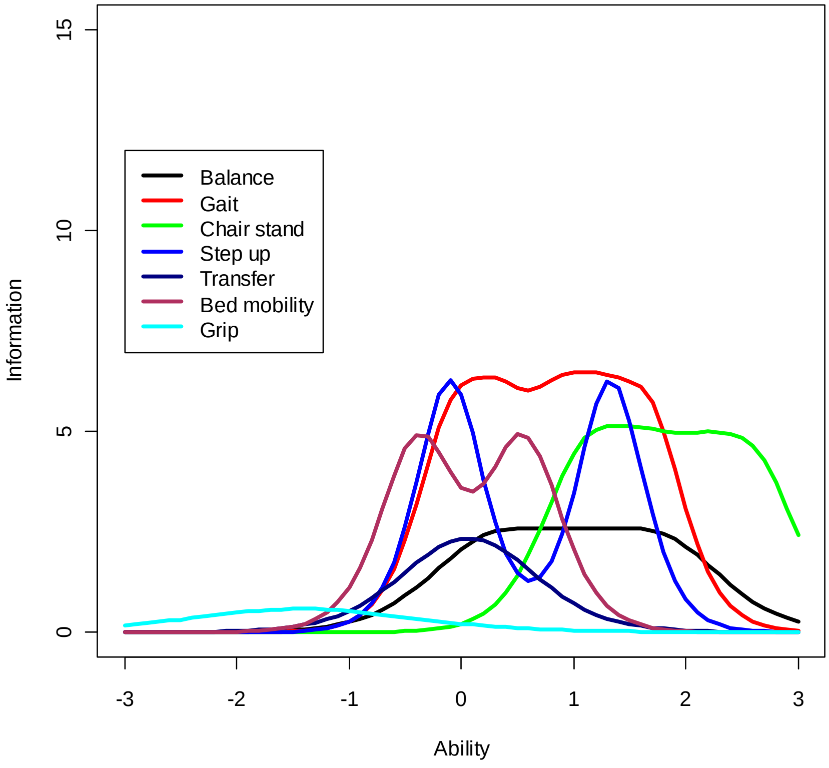

Figure S2

Scatterplot of GCS Composite Scores by Short Physical Performance Battery and Physical Performance Mobility Exam
